# ‘In silico labelling’: Exploring the biological landscape at the nanoscale using intelligent multimodal optical microscopy

**DOI:** 10.1111/jmi.70163

**Published:** 2026-08-31

**Authors:** A. Diaspro, P. Bianchini, R. Bizzarri, F. Cella Zanacchi, S. Civita, N. Incardona, L. Lanzanò, A. Morgantini, M. Salerno, L. Touijer

**Affiliations:** ^1^ Department of Nanoscopy, Italian Institute of Technology, CHT Erzelli Genoa Italy; ^2^ SEELIFE, Department of Physics University of Genoa Genoa Italy; ^3^ Department of Surgical Medical and Molecular Pathology, and Critical Care Medicine, University of Pisa Pisa Italy; ^4^ Department of Physics ‘E. Fermi’ University of Pisa Pisa Italy; ^5^ Department of Physics and Astronomy ‘Ettore Majorana’ University of Catania Catania Italy

**Keywords:** artificial intelligence, biophysics, fluorescence microscope, label free, macromolecular assembly, molecular biophysics, optical microscope, polarisation microscopy, STED microscopy, stimulated emission

## Abstract

Multimodal optical microscopy has recently converged with artificial intelligence (AI) to enable in silico labelling—the computational prediction of fluorescence‐like molecular contrast from label‐free measurements. Fluorescence plays a crucial role in linking microscopy and spectroscopy at molecular level, enabling image formation by means of linear and non‐linear investigation modalities. Over the past two decades, fluorescence optical microscopy has progressed into optical nanoscopy and single molecule localisation methods that operate at the nano‐ and even Ångstrom level under ambient conditions. Methods such as Stimulated Emission Depletion (STED), Photo‐Activated Localisation (PALM), Stochastic Optical Reconstruction (STORM), super‐resolution fluorescence lifetime imaging microscopy (FLIM) and image scanning microscopy (ISM), Minimal Fluorescence photon Flux (MINFLUX) microscopy allows us to investigate living cells at the molecular level. A significant and challenging development in this field is the coupling of fluorescence with label‐free polarisation and phase optical methods—for example, Mueller‐matrix (MM) microscopy—with the aim of extracting specific molecular information from label‐free datasets. In this review, we discuss how this convergence, together with modern generative modelling for in silico labelling, is turning the optical microscope into an intelligent instrument.

## 1 INTRODUCTION

Multimodal optical microscopy is significant because it allows to correlate structure, composition, processes and mechanics in the study of biological systems from multiple complementary perspectives, combining the strengths of different imaging methods within a single instrument or workflow.^1^, ^2^ It enables to integrate information at different spatial and temporal scales for studying complex biological network. In this scenario we will consider fluorescence^3^ and polarisation‐based^4^ label‐free as main methods to get image contrast. Fluorescence digital imaging microscopy is the well‐established bridge between microscopy and spectroscopy at the molecular scale:^5^ it enables image formation through linear^6^ and non‐linear^7^ modalities and provides environment‐sensitive observables—such as fluorescence lifetime^8^—that act as molecular stopwatches of biomolecular events.^9^, ^10^, ^11^ Over the past two decades, fluorescence optical microscopy has evolved into optical nanoscopy^12^, ^13^, ^14^ and single‐molecule localisation approaches,^15^, ^16^, ^17^, ^18^ operating at the nanometer and even at the Ångstrom scale under ambient conditions.^19^, ^20^ In parallel, advances in light sources, detectors, and computational processing have extended fluorescence imaging to the mesoscale while preserving 4D, *x*‐*y*‐*z*‐*t* capabilities.^10^, ^21^, ^22^, ^23^ The mesocale physical regime, between the molecular and cellular or tissue levels, is where also label‐free optical microscopy offers incredibly vast scenario^24^ is and includes quantitative phase imaging, scattering‐based and polarimetric methods.^25^, ^26^ Unfortunately, even if refractive index changes play a dominant role, label‐free techniques lack molecular specificity, which is an otherwise unique property of fluorescence labelling. In this context, artificial intelligence (AI) methods^27^ can play a key role to connect the optical properties of a sample to the molecular information available through super‐resolved fluorescence imaging.^28^, ^29^ The mesoscale is a biophysical realm where molecular interactions give rise to functional biological structures and emergent behaviours underlying physiological as well as pathological states of the cell/tissues setting. Worth mentioning are biophysical case studies such as chromatin organisation forming higher‐order domains, cytoskeleton networks including actin filaments, microtubules, and intermediate filaments assembling dynamically, membrane domains like lipid rafts or protein clusters regulating signalling, phase‐separated condensates including chromatin organisational motifs, and collective molecular processes related to mitochondria, Golgi, and ER shaping. In the case studies related to chromatin organisation,^30^, ^31^ one can consider, for instance, that in a single cell a fluorescently labelled nucleus^32^, ^33^, ^34^ and label‐free observations^35^, ^36^, ^37^, ^38^, ^39^, ^40^ can encode compaction information. Chromatin organisation and dynamics remain fascinating questions that can be explored and understood through optical microscopy.^41^, ^42^ The key is to learn the non‐trivial mapping between the two. Molecular‐rich images from label‐free observations may be framed as a supervised mapping between domains. In this study, we explore how modern generative modelling for in silico labelling can transform the optical microscope into an intelligent device. We present a two‐stage pipeline designed to generate specific molecular images from label‐free acquisitions.

## 2 FLUORESCENCE OPTICAL MICROSCOPY AT MOLECULAR SCALE

Optical microscopy techniques that harness fluorescence to overcome the diffraction limit are broadly referred to as ‘super‐resolved fluorescence microscopy’ (SR), also known as optical nanoscopy. Imaging can be performed in widefield, point scanning and image scanning configurations; in all cases, resolution gains arise from controlling, spatially and/or temporally, the excitation and/or the emission of fluorophores. Conceptually, SR approaches fall into two families: ‘coordinate‐targeted’ and ‘coordinate‐stochastic’ methods.^14^, ^43^, ^44^ The former encompasses scanning approaches that enhance resolution by optically engineering the point spread function (PSF) of the microscope without requiring computational steps. These techniques probe the specimen with a spatially modulated illumination source and include techniques such as Stimulated Emission Depletion (STED),^45^, ^46^ Reversible Reversible Saturable OpticaL Fluorescence Transition (RESOLFT).^47^ The latter refers to approaches where a sample image progressively emerges by stochastic reading‐out of sparse fluorophores, and is referred to as single‐molecule localisation microscopy (SMLM).^48^, ^49^, ^50^, ^51^ It includes widefield methods in which the resolution is improved by exploiting photoactivation, photoconversion, and ground‐state depletion of fluorescent molecules, for example, PhotoActivated Localisation Microscopy (PALM),^17^, ^52^, ^53^ Stochastic Optical Reconstruction Microscopy (STORM),^15^, ^54^ accumulated binding of diffusing probes (PAINT)^55^, ^56^ and Minimal Fluorescence photon Flux (MINFLUX).^57^, ^58^, ^59^, ^60^ Among targeted techniques STED microscopy is widely used in biological investigations because it can routinely deliver ∼ 30–40 nm *x*, *y* resolution on fixed and living samples. This method exploits the process of stimulated emission to arbitrarily ‘shrink’ down the PSF of the microscope, bypassing the diffraction limit. Recent implementations couple STED with image scanning microscopy (ISM)^61^ using SPAD‐array detectors^62^ and time‐resolved acquisition, exploiting single‐photon arrival times, to boost signal‐to‐noise and effective resolution. In this view, STED can be regarded as a tuneable, confocal‐style architecture enhanced by gated detection and detector‐array read‐out.^63^ Of note, STED is a platform exquisitely suited to imaging living cells and organisms. The single‐molecule localisation microscopy (SMLM) family improves resolution by exploiting the photoactivation, photoconversion, ground‐state depletion or transient binding events to isolate emitters in space and time. Localising these single emitters yields ∼ 5–30 nm precision laterally, while axial precision can be enhanced by introducing astigmatism (or other PSF engineering) in the detection path. Beyond imaging, SMLM enables quantitative analysis of the fluorophores in the specimen, such as local density estimation and cluster analysis. These exciting features make SMLM techniques a powerful approach to address biological processes and molecular organisation at an unprecedented resolution, albeit the slower acquisition pipeline has hampered the detection of fast‐diffusing biomolecules insofar. Yet, in the SMLM scenario, MINFLUX opens a new investigation window that can be clearly defined through two ways for its acronym, namely: ‘Minimal fluorescence photon fluxes’ and ‘Maximally informative luminescence excitation’.^57^ MINFLUX enables super resolution by localisation precision and real‐time tracking of single molecules, offering unmatched spatial and temporal resolution. Figure 1 reports super resolved imaging of microtubules compared with confocal imaging. Images are acquired with an Abberior MINFLUX microscope based on an Olympus IX83 equipped with a high numerical aperture objective (UPLXAPO 100×, NA 1.40) and exciting AlexaFluor647 (A647) fluorophores with a 640 nm laser.

**FIGURE 1:**
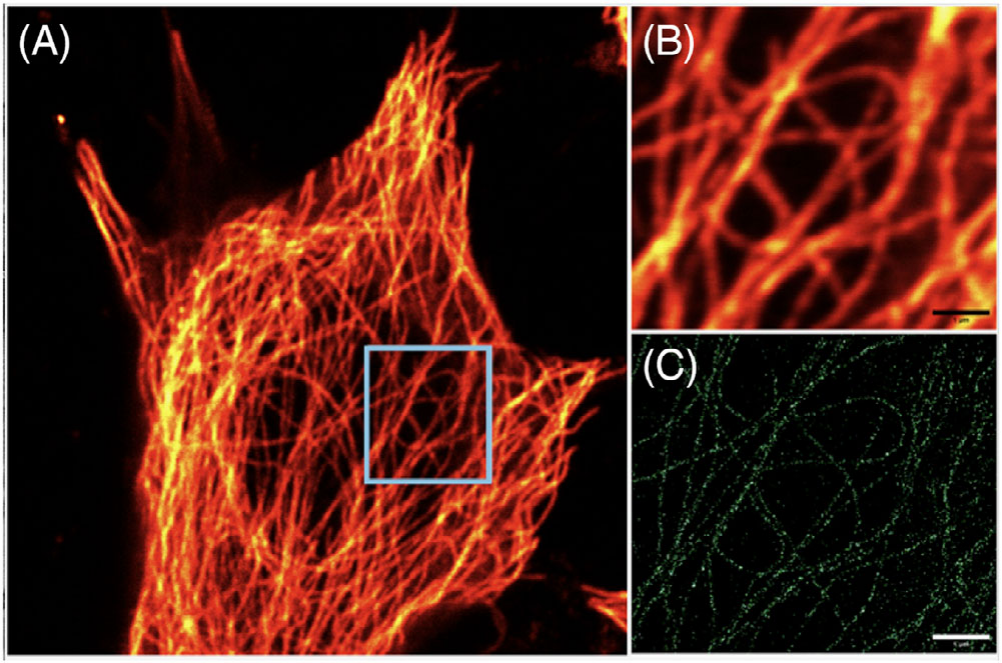
Microtubules MINFLUX imaging. Hela cells were fixed and labelled by indirect immunostaining using primary anti α‐tubulin and Alexa647‐labelled secondary antibody. (A) Whole cell confocal imaging of microtubules. (B) Confocal imaging of a selected region of interest (inset) and (C) corresponding MINFLUX image with nanoscale single‐molecule localisation precision. Scale bar: 5 µm (A) and 1 µm (B and C).

Mechanistically, MINFLUX localises a fluorophore by sequentially positioning an excitation field with a central intensity minimum (often implemented with Laguerre–Gaussian ‘doughnut’ modes) within a region of diameter L and recording the photon counts at each position. The relative counts encode the displacement from the minimum; maximum‐likelihood estimators recover the emitter position, and the Cramér–Rao bound (CRB) quantifies the theoretical lower limit of the localisation variance for a given photon budget, background, and geometry. In terms of spatial resolution, MINFLUX is wavelength and numerical aperture independent as illustrated in Figure 2.

**FIGURE 2:**
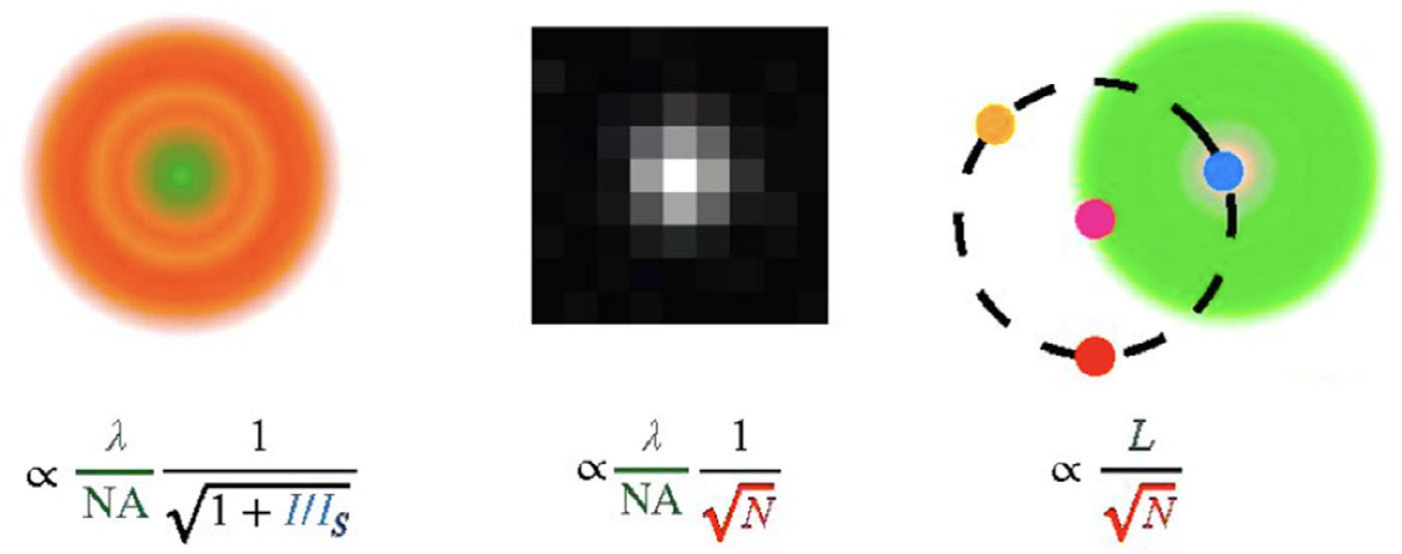
Parameters influencing spatial resolution for STED and localisation precision for ‘classical’ single molecule approaches and MINFLUX, from the left to the right.

The current technological drawback is that forming molecular‐scale images over whole‐cell fields remains time‐consuming. A pragmatic workflow acquires a fast confocal/ (gated)‐STED overview, then targets regions of interest for high‐precision MINFLUX. In this context, considering a two‐stage AI pipeline, an ESGRAN (Enhanced super‐resolution generative adversarial networks) network^64^ can be used  that is motivated precisely by the need to accelerate nanoscale analysis while preserving biological interpretability.^28^

## 3 LABEL‐FREE MUELLER‐MATRIX OPTICAL MICROSCOPY

A promising approach for the purpose of label‐free to fluorescence transformation is the one starting from the 16 images available with a Mueller matrix (MM) microscope.^25^, ^65^, ^66^, ^67^ Mueller matrix microscopy exploits the changes in the polarisation of light upon the interaction with matter,^25^, ^68^, ^69^ to achieve a label‐free signature of the sample. Using the Stokes‐Mueller formalism, the polarisation state of a light beam can be represented by the intensity of the 4‐element Stokes vector $S_{i}$. After the interaction with the sample, the polarisation state is described by a new Stokes vector, $S_{o}$, that is simply the multiplication of $S_{i}$ with the 4×4 MM of the sample, $\left[m_{i,j}\right]$, according to the relationship $S_{o}=\left[m_{i,j}\right]\cdot S_{i}$. Light polarisation is very sensitive to local physical anisotropies. In fact, as early demonstrated, MM microscopy can reveal structural changes in the sample down to the molecular scale.^38^ Moreover, the Mueller‐Stokes approach addresses changes in the polarisation state of light caused by known objects, such as the components of the microscopy system along the optical pathway to and from the unknown sample. For simplicity, we can think of the Mueller matrix (MM) as a fingerprint of the unknown sample, even though it includes all contributions from the optical pipeline entailed in the instrument calibration. In our case the MM carries information of the different biomolecular structures organised in the sample. $m_{14}$, for instance, describes circular dichroism. Operating outside the absorption bands of biological molecules of interest, specifically the ratio between $m_{14}$ and $m_{11}$, known as Circular Intensity Differential Scattering (CIDS), characterises chiral structures like chromatin.^70^ So far, CIDS is sensitive to the radius and pitch of these structures, which relate to their level of compaction.^36^ Other properties, such as those connected to changes in refractive index, can also be probed.^25^ An MM microscope is essentially built by adding a polarisation state generator on the illumination path and a polarisation state analyser on the detection path.^71^ Therefore, it can be built using both widefield and beam/point scanning architectures.^35^ High‐contrast MM imaging of single cells has been recently demonstrated through a darkfield scanning system.^72^ An example of the results obtained with this system is reported Figure 3 reporting about the MM label‐free signature (a) and the relationship between MM and fluorescence signature in the very same sample (b). In this case, the optical system is capable to retrieve six of the sixteen elements of the MM in a simple sample scanning, by means of an active polarisation modulator realised with a photoelastic modulator and lock‐in demodulation^73^ (Figure 3C). In Figure 3, fluorescence image has been captured with a commercial system (Nikon AX‐NSPARC), and the CIDS image, calculated as $CIDS\;=-m_{14}/m_{11}$. The architecture reported in Figure 3C can serve as a multimodal system for capturing MM‐fluorescence image pairs, enabling the training of a neural network to transform a label‐free image into a fluorescence image, in line with the two‐stage pipeline that incorporates molecular specificity and increased spatial resolution. Here, this is achieved by capturing the fluorescence signal in the backward pathway, utilising a commercial confocal scan‐head.^72^

**FIGURE 3:**
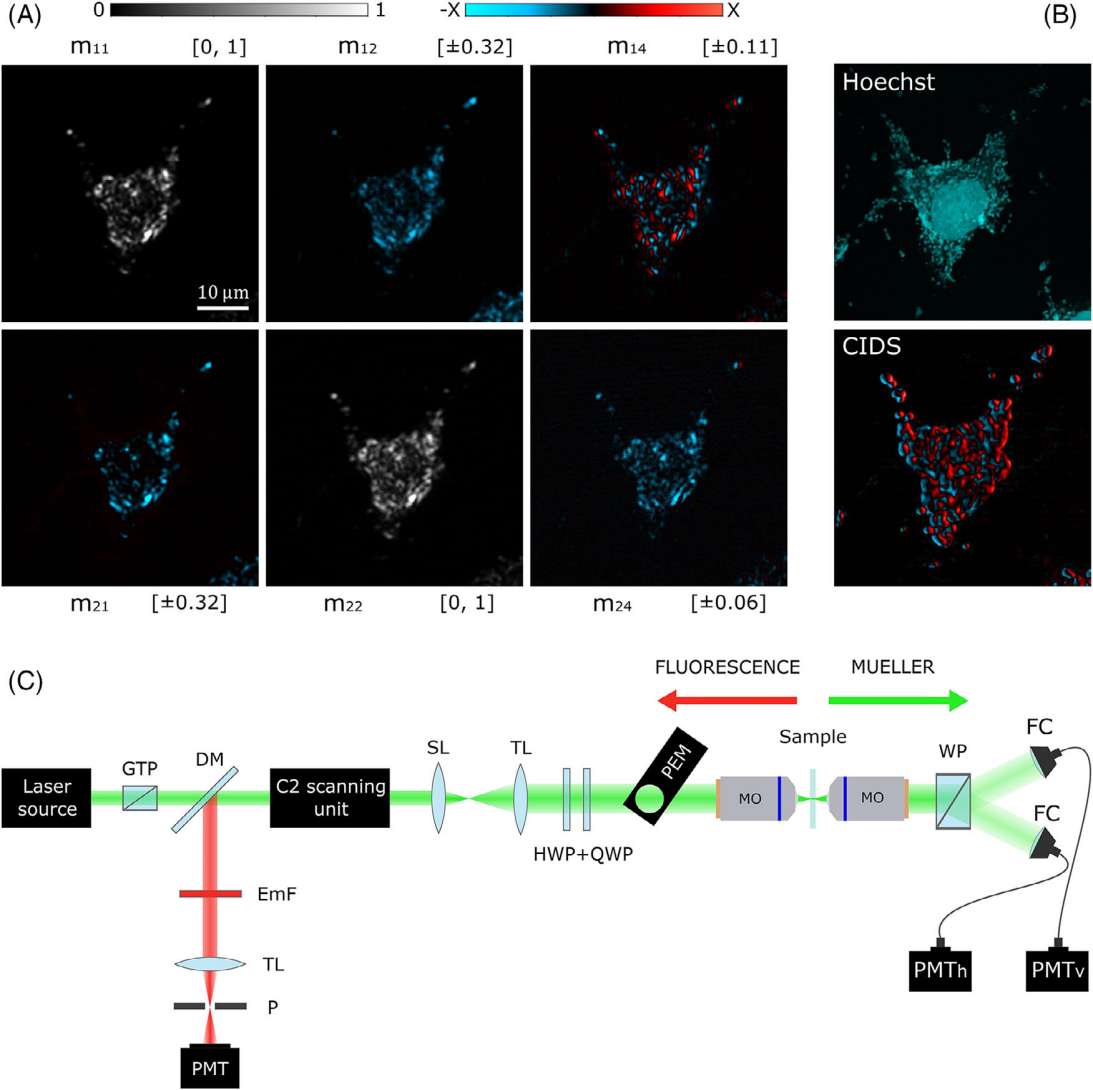
(A) The six images corresponding to the six elements of the Mueller matrix obtainable with a multimodal MM microscope. (B) The fluorescence image of a HeLa cell, stained with Hoechst for DNA labelling and the CIDS. (C) Multimodal MM‐fluorescence optical system. GTP stands for Glan–Thompson prism, DM for dichroic mirror, SL for scan lens, TL for tube lens, HWP/QWP for half/quarter waveplate, PEM for photoelastic modulator, MO for microscope objective, WP for Wollaston prism, FC for fibre coupler, PMT for photomultiplier tube, EmF for emission filter and P for pinhole.^72^

## 4 THE MULTIMODAL INTELLIGENT OPTICAL MICROSCOPE

Artificial intelligence is increasingly becoming a key component in the evolution of optical microscopy towards multimodal intelligent imaging systems. In this context, we chose conditional Generative Adversarial Networks (cGANs),^74^, ^75^, ^76^ a class of deep generative models designed to map between pairs of images from two different domains, by learning the underlying data distribution. cGANs provide a framework for a supervised image‐to‐image translation, and consist of two networks trained jointly: a generator and a discriminator. The generator, typically an encoder‐decoder like architecture, takes the label‐free image and learns to map to the corresponding fluorescence representation. The discriminator, a convolutional neural network, receives both real and generated fluorescence and learns to distinguish between them. Through this adversarial training, the discriminator encourages the generator to create increasingly realistic images, consistent with the true fluorescence images. To reach an intelligent multimodal microscopy, we adopt a two‐phase pipeline, illustrated in Figure 4, in which cGANs are used for modality translation and resolution enhancement. Importantly, each stage of the pipeline was independently validated using paired data specific to each modality, as a fully chained dataset covering all domains is currently unavailable.

**FIGURE 4:**
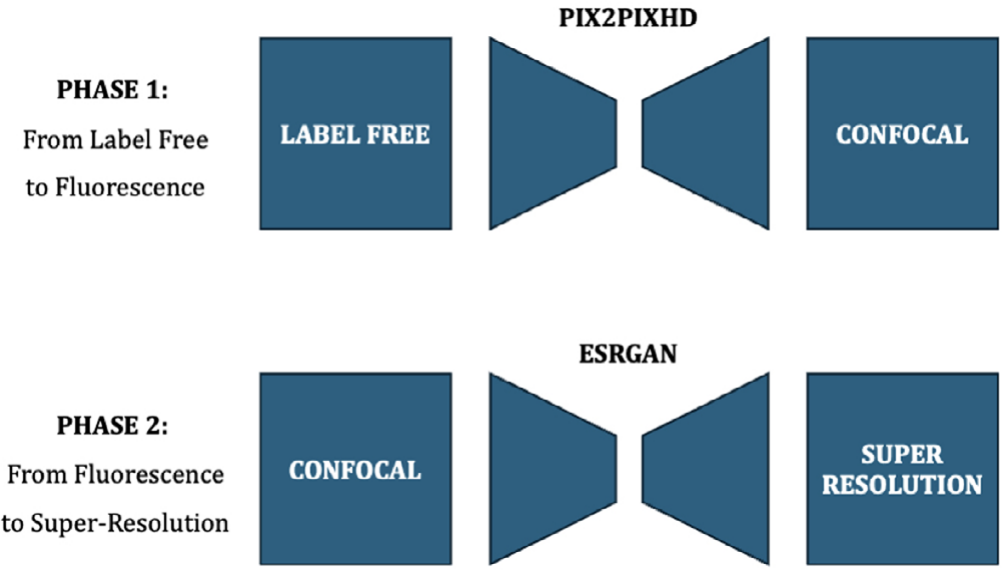
Two‐stage pipeline from label‐free to molecular imaging. In Phase 1, Pix2PixHD uses a label‐free image and generate the equivalent confocal fluorescence image; In Phase 2, ESRGAN takes a confocal image and generate the single molecule super‐resolution image.

For Phase 1 of our framework, we employed Pix2PixHD,^77^ a generative adversarial network tailored specifically for high‐resolution images synthesis, preserving the original microscope image dimensions. In addition to pixel level reconstruction loss, Pix2PixHD incorporates a features‐matching loss from the discriminator network, and a perceptual loss based on VGG deep neural network, to encourage the model to learn meaningful image representations while maintaining structural fidelity. We quantitatively evaluate the generated fluorescence images against the ground truth using Peak Signal to Noise Ratio (PSNR) and Structural Similarity Index Measure (SSIM),^78^ two standard metrics to assess generated images quality. PSNR reflects pixel‐wise fidelity through a Mean Square Error between the pixel intensities of the generated and the reference images, whereas SSIM evaluated the perceptual similarity in term of the mean intensity, variance and covariance. A dataset of 1000 paired images was used and partitioned into training, validation, and test subsets, with 20% reserved for testing and 20% of the remaining training data used for validation. The choice of the microscopy techniques to capture the label‐free and fluorescence image pairs depends mainly on the application. As an initial experimental configuration for the Phase 1, transmitted light was used to capture both the confocal and the corresponding brightfield image. Leveraging Pix2PixHD's ability to handle high resolution inputs, we train the model with the image's full dimensions (1024 × 1024). We follow the original training protocol, with 200 epochs, a batch size of 1, and a learning rate of 2e‐4. Similarly, data augmentation consisted of random flipping and rotations. The model achieved an average PSNR of 24.4 dB and SSIM score of 0.82 across the test set. Figure 5 illustrates an example of a Label‐Free source image, the corresponding Fluorescence and the generated Fluorescence by our trained Pix2PixHD.

**FIGURE 5:**
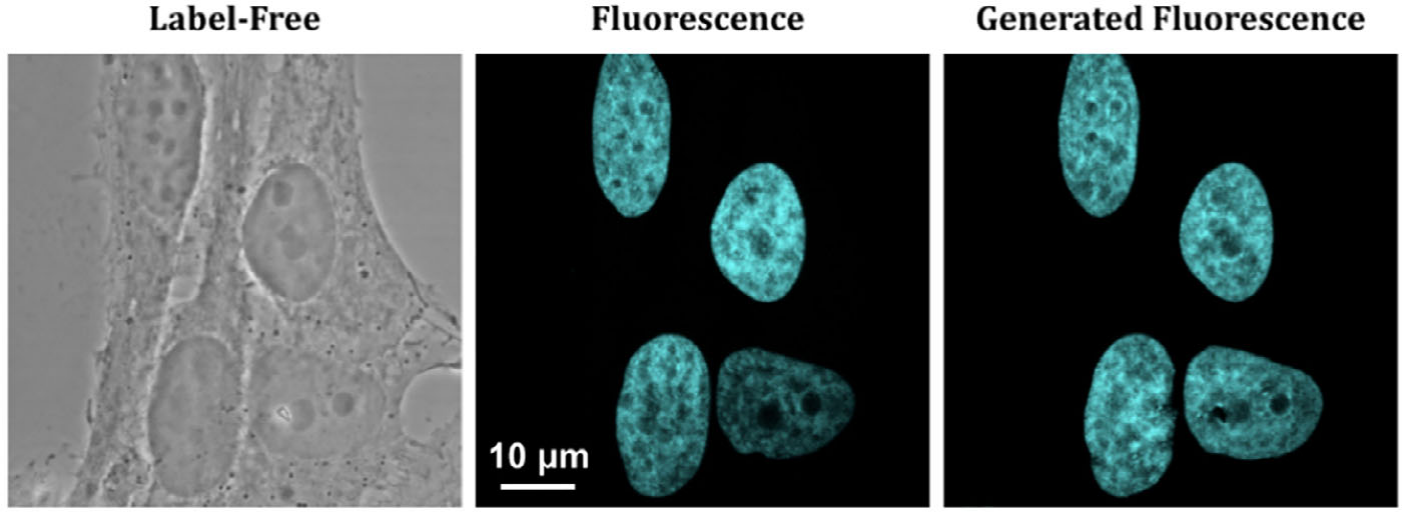
Example of images of the dataset used for Phase 1 based on a label‐free (left) and fluorescence (centre) image pair. On the right, the result of the label‐free to fluorescence transformation through Pix2PixHD. Images acquired using a Nikon A1 Eclipse microscope equipped with a CFI Plan Apo DM Lambda 100X Oil MRD31905.

Building upon Phase 1 where Pix2PixHD was used to translate label‐free images into fluorescence representations, Phase 2 focuses on recovering fine molecular details. The available fluorescence microscopy techniques encompass a wide range of methods that can resolve more details, reaching down to the molecular level, compared to label‐free techniques. Visually speaking, we aim to transform a cluster of molecules, which appear as a single blob, into individual ‘dots’ endowed with single‐molecule information, as shown, for example, in Figure 1. From a generative models perspective, this task can be formulated as Super Resolution (SR), where the generative model learns to upscale a lower resolution input image to a higher resolution, while preserving the semantic and structural details. In our case, the SR model is trained to transform the low details confocal image, into a clear super‐resolved image of the molecular structure. However, a substantial resolution gap exists between a confocal fluorescence microscopy and a single molecule imaging technique like MINFLUX, that an SR generative model would struggle to learn a direct transformation. A starting point is to introduce an intermediate resolution level, such STORM, allowing the generative model to gradually learn to retrieve more details out of the Confocal image. To implement this strategy, we employ Enhanced Super Resolution GAN (ESRGAN),^28^, ^64^ a well‐established super resolution model originally developed for natural images, yet adaptable for our specific task. The goal is not simply increasing image dimensions, but to learn meaningful characteristics of the images. ESRGAN is designed to learn meaningful features by means of combinations of the perceptual loss, that compares the features of the real and generated image, together with a discriminator that evaluates the realism of the generated images. Figure 6 shows how ESRGAN can be used starting from a confocal image to generate its super resolution version in the perspective of gradually enabling the transformation confocal → STORM → MINFLUX.

**FIGURE 6:**
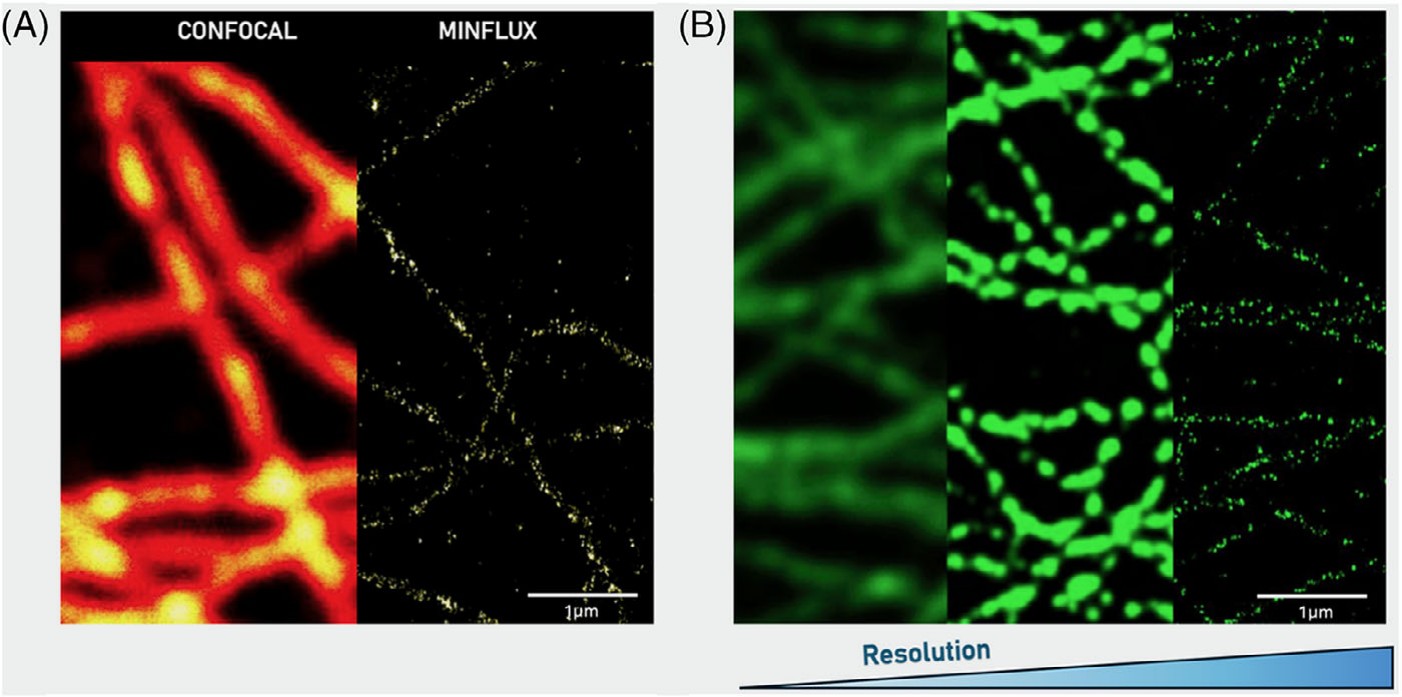
In HeLa cells α‐tubulin was labelled with indirect immunostaining using Alexa647 and imaged (A) by confocal (left) and MINFLUX microscopy (right). Resolution improvement (B) provided by AI‐assisted super‐resolution microscopy from confocal imaging (left), AI‐generated super‐resolution STORM‐like image (centre) and ‘true’ MINFLUX image (right), which is the target result of new AI‐generative models from confocal images.^28^

## 5 CONCLUSION

In conclusion, we have reviewed scientific and technical steps related to our research activities in the last 25 years that we are assembling towards the development of a multimodal optical microscopy platform empowered by artificial intelligence. Such a platform through an ‘in silico labelling’ allows the exploration at the nanoscale of molecular landscapes within the biological cell. It is an intelligent microscope, as it integrates a combination of fluorescence, label‐free, and polarisation‐ or mechanics‐sensitive modalities, globally achieving enhanced spatial resolution, quantitative accuracy, and temporal efficiency. Artificial intelligence is introduced as a new component of the optical microscope in a scenario that offers incredibly advanced illumination, detection and computation developments. Beyond the reported implementation, the methodology can be generalised to a broad range of imaging modalities, including nonlinear, interferometric, and scattering‐based techniques, creating a unified ‘liquid’ framework for data‐centric imaging at multiple scales.^79^, ^80^ An important limitation is the absence of a single dataset containing matched label‐free, confocal, and super‐resolution acquisitions of the same specimen. Future work will focus on acquiring such multimodal datasets to enable full end‐to‐end validation of the proposed pipeline. It is a matter of fact that the synergy between light and artificial intelligence opens the way towards artificial microscopes capable of learning from biological and material samples to enhance contrast, minimise photodamage, and reveal hidden correlations across structural and functional dimensions. Such systems will not only advance the frontiers of biophotonics and nanoscopy but also redefine the interaction between human insight and machine intelligence in scientific discovery.

While PSNR and SSIM provide quantitative measures, they do not directly assess biological validity of generated structures. Evaluating synthetic images using task‐based metrics or biologically meaningful descriptors would provide additional insight into their utility for scientific analysis. Additionally, such evaluations require task‐specific ground‐truth that are not currently available for our dataset. Future work will therefore focus on integrating biologically informed validation protocols to further assess the reliability of generated images for downstream quantitative analysis.
